# Inhibition of pSTAT1 by tofacitinib accounts for the early improvement of experimental chronic synovitis

**DOI:** 10.1186/s12950-019-0206-2

**Published:** 2019-01-29

**Authors:** Sandra Pérez-Baos, Paula Gratal, Juan I. Barrasa, Ana Lamuedra, Olga Sánchez-Pernaute, Gabriel Herrero-Beaumont, Raquel Largo

**Affiliations:** 1 Bone and Joint Research Unit, Rheumatology Department, IIS-Fundación Jiménez Díaz UAM, Avenida Reyes Católicos, 2. 28040, Madrid, Spain; 2 Department of Molecular Biology, Umeå University, 901 87 Umeå, Sweden

**Keywords:** Rheumatoid arthritis, Synovitis, Janus kinase inhibitors, Tofacitinib

## Abstract

**Background:**

In order to gain insight into the early effects drawn by JAK inhibitors on intra-joint JAK/STAT-dependent signaling, we sought synovial activation of STATs and their end-products, along with their modification with tofacitinib (TOFA), at flare-up in antigen induced arthritis (AIA). New Zealand rabbits were randomly assigned to four groups –healthy controls, AIA, TOFA-treated AIA, or TOFA-treated controls–. AIA was induced with 4 weekly intra-articular ovalbumin injections in sensitized animals. TOFA (10 mg·kg^− 1^·day^− 1^) was administered for the last 2 weeks. Animals were euthanized 24 h after the last injection.

**Results:**

AIA animals showed high-grade synovitis, which was partially improved by TOFA. No effects of the treatment were found on serum C-reactive protein or on the synovial macrophage infiltration at this stage. Synovial MMP-1,-3 and -13 expression levels in treated AIA rabbits were found to drop to those of controls, while a downregulation of IL6, IFNγ and TNF was evident in treated versus untreated AIA rabbits. Concurrently, a reduction in pSTAT1 and SOCS1, but not in pSTAT3, SOCS3 or active NFκB-p65, was noted with TOFA.

**Conclusions:**

Studying the mechanism of action of immunomodulatory drugs represents a major challenge in vivo, since drug-dependent decreases in inflammation very likely mask direct effects on disease mechanisms. This study design allowed us to prevent any confounding effect resulting from reductions in the overall inflammatory status, hence assessing the true pharmacological actions of TOFA in a very severe synovitis. Our findings point to pSTAT1 and MMPs as early molecular readouts of response to this JAK inhibitor.

**Electronic supplementary material:**

The online version of this article (10.1186/s12950-019-0206-2) contains supplementary material, which is available to authorized users.

## Background

The synovial membrane is the primary site of inflammation in rheumatoid arthritis (RA). It is organized as an interstitial tissue overlaid by an intimal lining of 1 to 3 rows of cells. These are named type A or type B synoviocytes, depending on their respective macrophagic or fibroblastic lineage, while the subintima is rich in collagen fibers, adipocytes, fibroblasts, blood vessels and nerves. The resting synovium becomes activated during RA, and is transformed into an invasive tissue, which eventually destroys joint cartilage and subchondral bone [1–3]. Pro-inflammatory cytokines such as tumor necrosis factor (TNF), interleukin (IL)1β, IL6 and interferon gamma (IFNγ) are acknowledged drivers of rheumatoid synovial transformation –or synovitis– as also of RA-associated systemic features. In rheumatoid synovitis these soluble mediators exert pleiotropic functions, including production of metalloproteinases (MMPs), generation of reactive oxygen species, and attraction of immune cells. They also promote angiogenesis and help increase cell survival [4–6].

Activation of the Janus kinase (JAK) and Signal Transducer and Activator of Transcription (STAT) pathway by type I/II cytokines is currently regarded as a major contributor to the sustained inflammatory response of RA [7, 8]. Accordingly, this pathway has become a promising target of RA therapeutics [9]. The JAK family of tyrosine kinases comprises 4 members, JAK1 to 3, and Tyk2, each of which can associate with specific cytokine receptors, and lead to the recruitment and activation of STATs. There are 7 isoforms of STATs –namely STAT1 to 6, with STAT5 subtypes A and B– which display a cell and tissue dependent distribution and account for the production of cytokines, proteases and growth factors in response to environmental stressors [8].

The JAK/STAT signaling pathway is tightly regulated by Suppressor of Cytokine Signaling (SOCS) proteins, whose transcription is in turn elicited by STATs. These proteins act as negative feedback inhibitors through three different mechanisms: direct inhibition of JAK activity, competition with STATs for the substrate or ubiquitination and proteasome-mediated degradation of JAKs and their coupled cytokine receptors [10]. At the end of the stimulation, these negative regulators undergo proteasome degradation [11]. Evidence points to a RA-associated upregulation of both SOCS1 and SOCS3 in certain cell types –such as synovial macrophages and peripheral blood mononuclear cells– [12]. While several authors have outlined these negative feedback inhibitors as an attractive strategy for RA therapeutics [13–15], there are no studies so far showing the effects of current treatments on these regulatory proteins.

Tofacitinib (TOFA) is a JAK inhibitor approved for the treatment of RA, which shows high JAK1/3 and little JAK2 affinity, while its effect on Tyk2 is negligible [16]. In patients with background methotrexate, TOFA resulted in the downregulation of IFNγ-induced chemokines at the synovium already after 1 month of treatment. Nonetheless, it did not change the expression of pro-inflammatory cytokines, nor did it improve histopathology at this time [17]. In the same study, a decrease in synovial pSTAT1 and 3 levels predicted a good clinical response at 4 months.

Even though the synovium is the main target of the disease, the ability of TOFA and other JAK inhibitors to modulate the cascade of pathogenic events at the joint in the short term has not been explored further. In addition, while the selectivity of these inhibitors for JAK subtypes has been broadly studied, little is known on their predominant effects on particular STATs and their downstream network of cytokines, regulatory factors and other related pathways. In fact, although an effect of TOFA on the canonical NFκB pathway has been previously described [18, 19], it is not clear whether in vivo these effects are due to a direct interaction between these two pathways [20, 21] or are rather linked to a secondary phenomenon related to the downregulation of NFκB-activating cytokines after JAK/STAT blockade. Additionally, effects of TOFA on the immunosuppressive and pro-regenerative cytokine IL-10, whose gene expression is known to be downregulated by IFNγ [22], are very few and controversial [18, 23, 24].

Precisely, the evaluation of early synovitis and its modification with therapy is a major limitation of human studies, since specimens are usually collected at the time of joint replacement surgery, therefore reflecting long-standing disease, and often coming from patients with a list of past and present medications. In this regard, animal models stand as an attractive alternative to explore mechanisms directly targeted by drug interventions in the rheumatoid joint, instead of those effects resulting from dampening inflammation.

In a rabbit model of chronic antigen induced arthritis (AIA) which faithfully reproduces rheumatoid synovitis, we studied the earliest modifications drawn by TOFA at joints during flare-up reactions, in order to explore the changes induced by this inhibitor on phosphorylated STATs and their end products in the short term.

## Methods

### Animal model

**Fig. 1:**
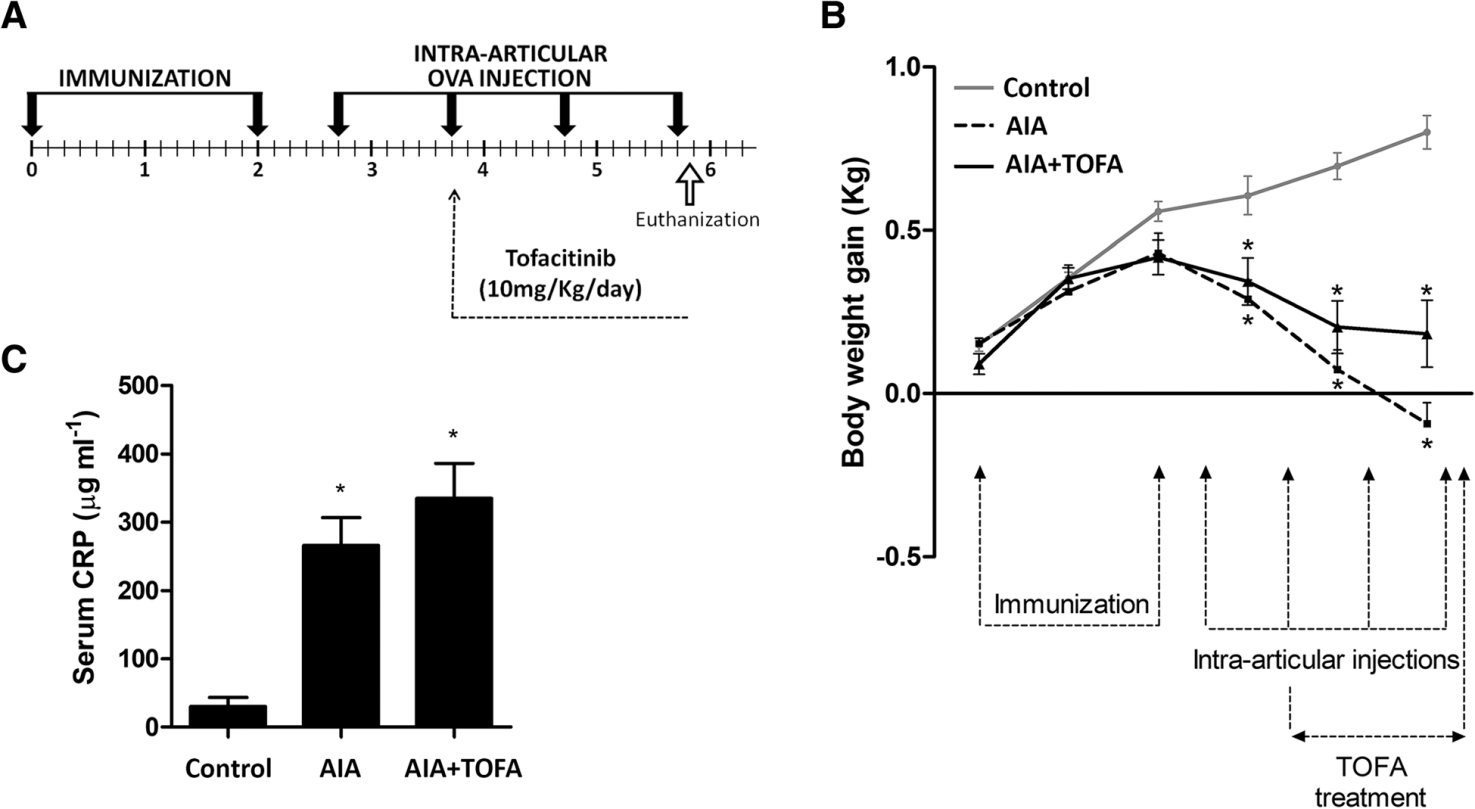
Systemic alterations in a rabbit model of antigen induced arthritis (AIA). **a.** Chronogram of the experimental model. **b.** Body weight gain of rabbits throughout the study **c.** Concentration of C-Reactive protein in serum. Data is shown as the mean and SEM (*n* = 8 rabbits per group).*p < 0.05 vs. Control. AIA: antigen induced arthritis; CRP: C-Reactive protein; TOFA: tofacitinib

Twenty-four New Zealand white male rabbits, 3 months old, with a weight of 2.6–3.0 kg (Granja San Bernardo, Navarra, Spain) were acclimatized and assigned to any of the following groups: healthy controls (Control, *n* = 8), placebo-treated AIA (AIA, *n* = 8) and TOFA-treated AIA (AIA + TOFA, *n* = 8). Animals were maintained on an ad libitum diet of commercial chow and water. Antigen-induced arthritis was carried out with the injection of 5 mg ml^− 1^ ovalbumin (OVA, Sigma-Aldrich, St Louis, MO, USA) into each of the knee joints of previously immunized rabbits. Intra-articular injections were thereafter repeated weekly (*n* = 4) in order to elicit a severe phenotype characterized by chronic rheumatoid-like synovitis with acute flare-up relapses as described [25, 26]. Controls received weekly intra-articular saline injections instead of the antigen. Treatments, consisting of TOFA (10 mg kg^− 1^ day^− 1^) or placebo, were administered as previously described [25, 27–29] *via* oral gavage from the time of the second intra-articular injection until euthanization, 15 days later (Fig. 1 a). Four additional healthy rabbits were treated with TOFA (TOFA, *n* = 4). The last dose of TOFA/placebo was given at disease flare-up, 1 day after the fourth intra-articular injection and 4 h before euthanization. This scheme of disease and treatment not only mimics the flares of human disease, but also allows the study of the very early and direct effects of the treatment in the synovium.

Body weight was weekly assessed throughout the study. The rabbits were euthanized after an 8-h fasting period with intra-cardiac pentobarbital (50 mg kg^− 1^; Tiobarbital, Braun Medical SA, Barcelona, Spain). At end points, blood samples were drawn from the marginal ear vein, and serum was collected and stored at − 80 °C until use. The knee synovial tissue was excised and cut into 2 equal pieces to be employed for histopathology and molecular studies, respectively.

Both the animal care and the experimental protocols of the study complied with the Spanish Regulations and UE Guidelines for the Care and Use of Laboratory Animals and were approved by our Institutional Review Board for Research (Ref. 2013/10).

### Determination of CRP in rabbit serum

C-reactive protein (CRP) levels were measured with a specific commercial enzyme-linked immunosorbent assay (ab157726, Abcam, Cambridge, UK), following manufacturer’s instructions, as described [25].

### Histopathology

Tissues were fixed in 10% formalin for 24 h, dehydrated and paraffin embedded. A blinded evaluation of histopathology was done in 4 μm hematoxylin-eosin stained sections using a standardized scoring method as previously described [30, 31]. Briefly, lining hyperplasia, fibrovascular alterations at the interstitium, and the tissue infiltration with leukocytes were independently assessed in 0 to 3-point semiquantitative subscales, and the global score was calculated with the sum of the subscales up to a maximum of 9 points.

### Synovial macrophage immunostaining

Synovial macrophages were identified with immunostaining techniques as previously described [25]. Briefly, 4 μm tissue sections were deparaffinized, hydrated in graded ethanol and incubated in 4% bovine serum albumin (BSA) and 3% sheep serum to block unspecific immunobinding. A monoclonal mouse anti-rabbit macrophage antibody (RAM11, 36.2 mg L^− 1^, Dako, Glostrup, Denmark) was added overnight, 4 °C. The binding was detected using biotinylated goat anti-mouse immunoglobulin G (IgG) (GE Healthcare, Little Chalfont, Buckinghamshire, UK) and peroxidase ABC with 3,3 diaminobenzidine tetra-hydrochloride as chromogen (Dako, Golstrup, Denmark). Sections were counterstained with hematoxylin, mounted in DPX medium (VWR International, Leuven, Belgium) and photographed using an automated iScan Coreo slide scanner (Ventana Medical Systems, USA). Five random areas per slide were selected for a blinded evaluation and quantified with Image J software as previously described [25]. Area selection relies on the following criteria: 1) both lining and sublining are well represented, 2) areas with blank spaces are excluded, 3) areas with artifacts are excluded. Results were expressed as the percentage of positive stained area.

### RNA isolation and RT- PCR

RNA was isolated using TRIzol reagent (Roche Diagnostics, Barcelona, Spain), dissolved in nuclease-free water and quantified with a NanoDrop ND1000 spectrophotometer (Thermo Fisher Scientific, Waltham, MA, USA). cDNA was obtained from 1 μg of total RNA using the High Capacity cDNA Reverse Transcription Kit (Applied Biosystems, Foster City, CA, USA) following manufacturer’s instructions. RNA expression was quantified by single-reporter real-time PCR using the Step One Plus Detection system (Applied Biosystems, Foster City, CA). Specific TaqMan probes for rabbit TNF, IL1β, IL6, IL10, IFNγ, MMP-1, MMP-3 and MMP-13 were purchased from Applied Biosystems. Gene expression levels were calculated with the ΔΔCt method using 18S as internal control.

### Preparation of total and nuclear extracts from tissue

Frozen minced synovial tissues were pulverized in liquid nitrogen, and 50 mg were homogenized for protein extraction in 15 mM HEPES, 10% glycerol, 0.5% NP-40, 250 mM NaCl, 1 mM EDTA, 1 mM PMSF, 1 mM NaF, 1 mM β-glycerophosphate, 1 mM Na_3_VO_4_ and protease-inhibitor cocktail (Sigma-Aldrich, St. Louis, Missouri, USA). Extracts were centrifuged (15 min, 16.000 x g, 4 °C). Supernatants were collected, and protein concentration was measured with BCA Protein Assay Kit (Pierce, Rockford, IL, USA).

Nuclear extracts were isolated from 50 mg of pulverized tissues, after homogenization in 20 mM HEPES, 5 nM NaF, 10 μM Na_2_MoO_4_, 0.1 mM EDTA, 0.01% NP-40, 1 M ditiotreitol (DTT) and a phosphatase- and protease-inhibitor cocktail (Sigma-Aldrich, St. Louis, Mo, USA). Extracts were centrifuged at 4 °C, 10 min, 850 x g. Cell pellets were incubated in hypotonic buffer, 15 min, at 4 °C, and centrifuged 30 s, 22000 x g, 4 °C. Nuclear pellets were then eluted in 50 μl of Complete Lysis Buffer (Active Motif, La Hulpe, Belgium), incubated 30 min on ice on a rocking platform and centrifuged 10 min, 22,000 x g, 4 °C. Supernatants were collected and protein concentration was determined using Bradford’s method (Bio-Rad, Madrid, Spain).

### Western blotting

30 μg of total cell lysates were subjected to electrophoresis in SDS-polyacrylamide gels, at 100 V, for 2 h and then transferred to nitrocellulose membranes in a semi-dry Trans-Blot device (Bio-Rad, Madrid, Spain) for 30 min at 25 V. Membranes were blocked in 3% skimmed milk, 1 h, RT, and incubated with antibodies against pSTAT1 (5 μg ml^− 1^, 14–9008, Affymetrix eBioscience, SanDiego, CA, USA), pSTAT3 (2.5 μg ml^− 1^, MAB4607, R&D Systems, Minneapolis, MN, USA), SOCS1 (1 μg ml^− 1^, ab9870, Abcam, Cambridge, UK) and SOCS3 (0.73 μg ml^− 1^, ab119806, Abcam, Cambridge, UK). Antibody binding was detected with chemiluminescence using peroxidase-linked species-specific secondary antibodies from GE Healthcare (Little Chalfont, Buckinghamshire, UK). Coomassie blue staining (G1041, Sigma-Aldrich, St Louis, MO, USA) was employed as loading control and densitometric units were normalized by the average value of the healthy controls, as a chemiluminescence reference to compare blots.

### Measurement of NFκB-p65 activation by DNA-binding enzyme linked immunosorbent assay (ELISA)

The nuclear activation of NFκB-p65 was determined in 5 μg of nuclear protein with the TransAM NFκB-p65 ELISA kit (40,096, Active Motif, La Hulpe, Belgium), according to the manufacturer’s instructions. Absorbance was measured at 450 nm.

### Statistical analysis

Data were expressed as mean ± SEM and analyzed with non-parametric tests. Kruskal-Wallis test and Dunn’s post hoc test were run for multiple group comparisons, while two-group comparisons were done with Mann-Whitney U for independent samples, respectively. A *p*-value of less than 0.05 was considered significant. Statistical analysis was performed using Windows SPSS 21.0 (SPSS, Inc., Chicago, IL, USA).

## Results

### Effect of TOFA on serum C-reactive protein and body weight gain of AIA rabbits

The high inflammatory status evoked by the experimental disease led to a decreased weight gain, already evident in arthritic animals from the first intra-articular challenge and continuing thereafter until the end of the study (Fig. 1 b). This effect was not so pronounced in the TOFA-treated AIA group, with a difference between both groups approaching significance (*p* = 0.07) (Fig. 1 b). At the time of the evaluation, there was also a substantial increase in serum CRP in AIA animals which was not decreased by TOFA treatment (Fig. 1 c). TOFA did not have any effect on healthy rabbits (data not shown).

### Synovial histopathology and macrophage infiltration

**Fig. 2:**
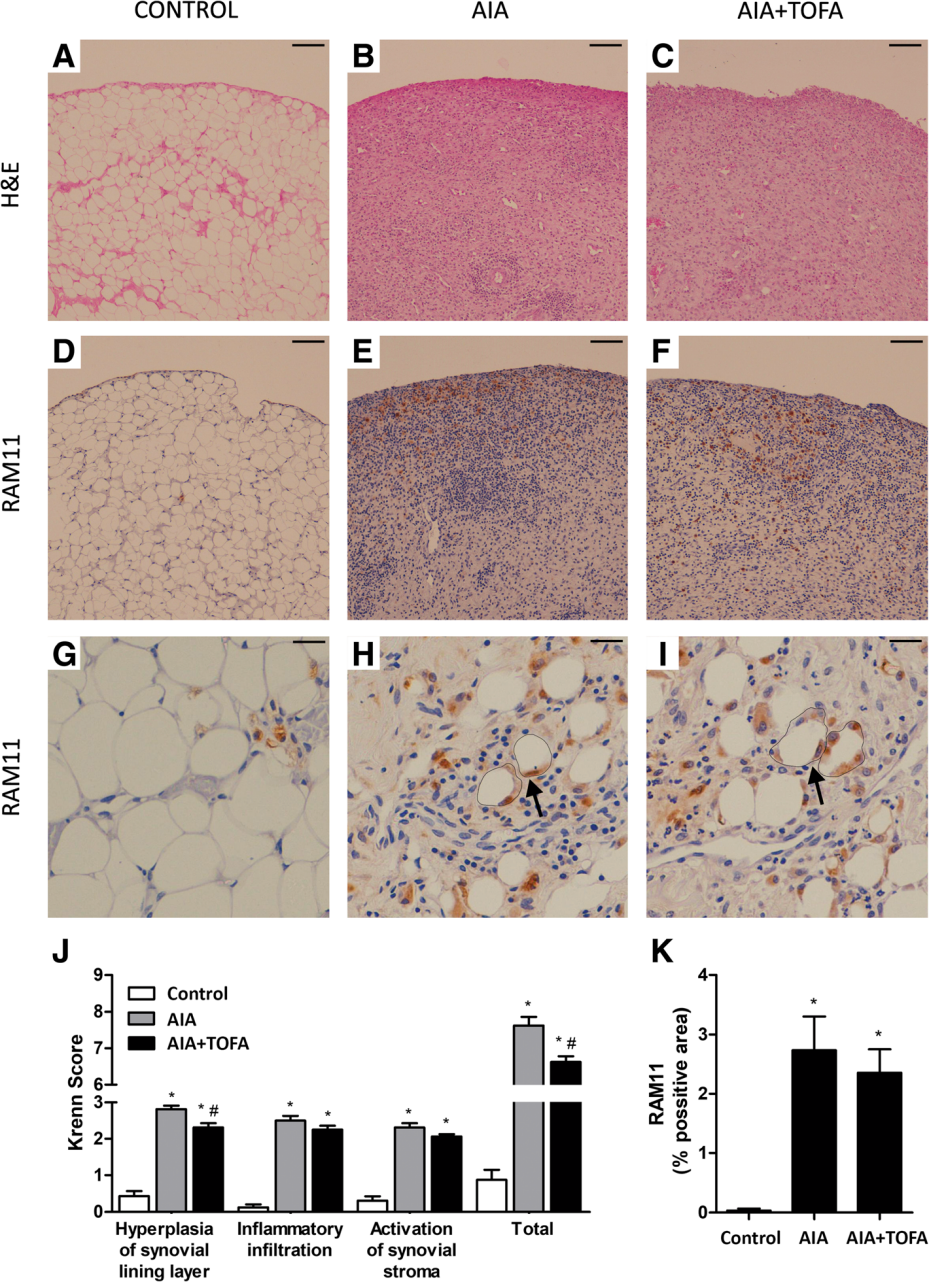
Assessment of synovitis and macrophage infiltration. **a**-**c**. Representative sections of the synovium stained with hematoxylin and eosin. Control (**a**), AIA (**b**) and AIA + TOFA (**c**). Scale bar = 100 μm. **d-i.** Immunohistochemistry of representative sections of the synovium using the monoclonal anti-rabbit macrophage antibody RAM11. Control (**d**, **g**), AIA (**e**, **h**), AIA + TOFA (**f**, **i**). Scale bar (**d**-**f**) = 100 μm. Arrows point to representative crown-like structures (**h**, **i**) which are surrounded by a line, scale bar (**g**-**i**) = 25 μm. **j**. Global synovitis score. Bars show the mean and SEM (*n* = 16 paws per group). *p < 0.05 vs. Control, #*p* < 0.05 vs. AIA. **k.** Densitometric analysis of RAM11 staining percentage in the synovium of each group of animals. Data is shown as the mean and SEM (*n* = 16 paws per group).*p < 0.05 vs. Control*.*AIA: antigen induced arthritis; TOFA: tofacitinib

The AIA group showed a high grade synovial inflammation, characterized for an increased thickening of the synovial lining, a widespread infiltration with inflammatory cells, which included lymphoid aggregates, and an enlarged and hypercellular stroma. The AIA + TOFA group had a significantly lower lining hyperplasia and they also showed slightly, albeit not significantly, better scores in the inflammatory infiltration and in the stromal hypertrophic changes. As a result, global scores were partially but significantly improved by the treatment (Fig. 2 a–c and Fig. 2J).

In both treated and non-treated AIA rabbits, RAM11 immunohistochemistry not only revealed an increase in intimal macrophages but also a macrophage infiltration at the synovial interstitium, where they frequently acquired a crown-like disposition surrounding adipocytes (Fig. 2 d–i, arrows). TOFA did not produce any perceptible changes in the amount of these cells nor did it alter their disposition at the subintimal layer (Fig. 2 k). TOFA treatment did not have any effect on healthy rabbits (data not shown).

### Expression of anti and pro-inflammatory genes and MMPs in the synovium

**Fig. 3:**
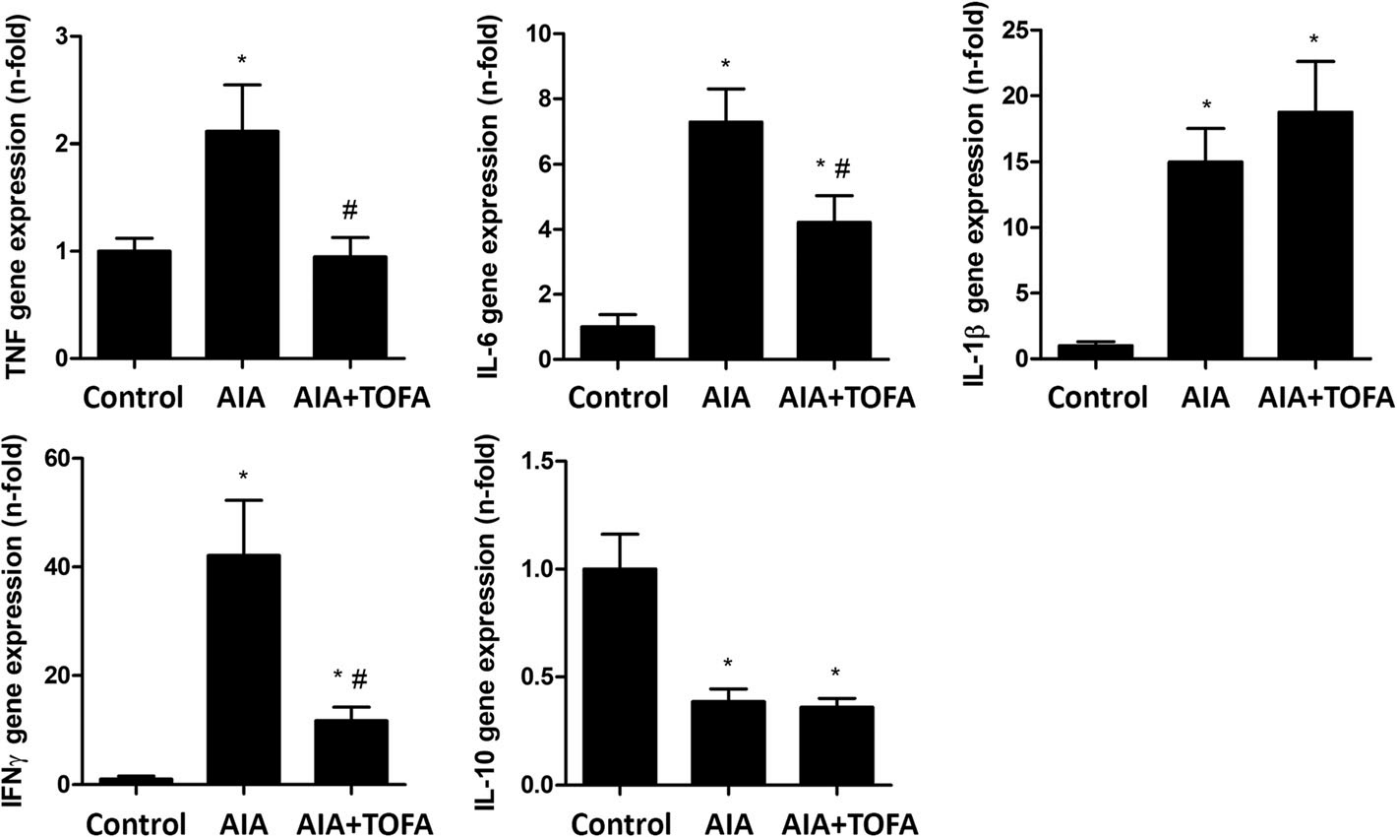
Gene expression of pro and anti-inflammatory factors in the synovium of AIA rabbits after TOFA treatment. Gene expression analysis of TNF, IL6, IL1β, IFNγ and IL10 in the rabbit synovium. Data is shown as the mean and SEM (*n* = 16 paws per group). * *p* < 0.05 vs. Control, # *p* < 0.05 vs. AIA. AIA: antigen induced arthritis; TOFA: tofacitinib; TNF: tumor necrosis factor; IL: interleukin; IFN: interferon

We next evaluated early induction of the gene expression of cytokines and MMPs in the synovium of AIA rabbits, either with or without TOFA treatment. There was an increase in gene expression levels of TNF, IL1β, IL6 and IFNγ, along with a downregulation of IL10 in AIA rabbits as compared to controls. Of note, TOFA treatment restored TNF gene expression to that shown by control rabbits. Similarly, treated rabbits had comparatively lower levels of IL6 and IFNγ than untreated AIA rabbits, while there were no differences between both groups in the gene expression levels of IL1β and IL10 (Fig. 3).

**Fig. 4:**
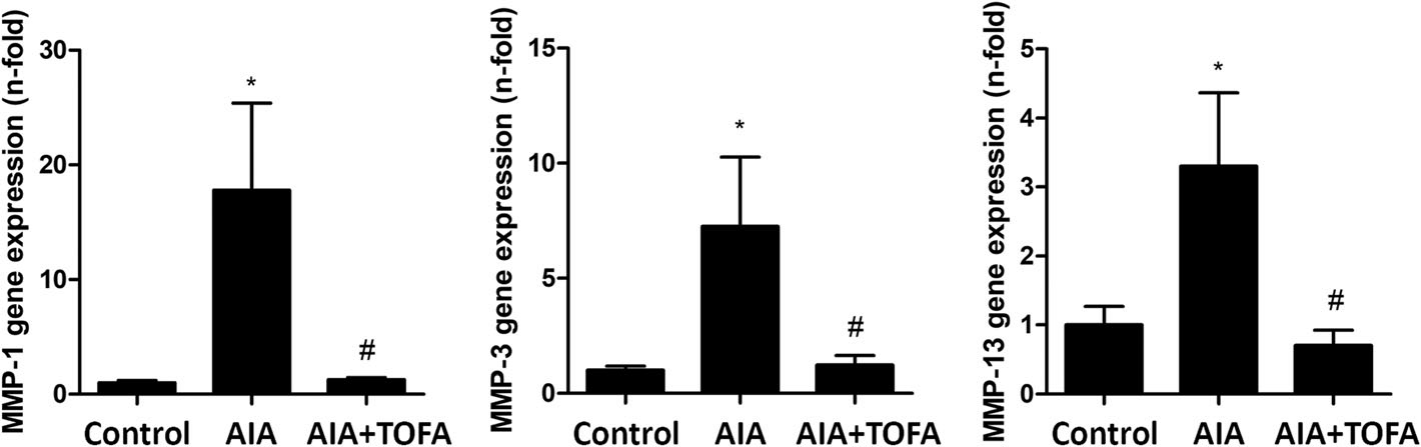
Gene expression of metalloproteinases in the synovium of AIA rabbits after TOFA treatment. Gene expression analysis of MMP-1, MMP-3 and MMP-13 in the rabbit synovium. Data is shown as the mean and SEM (*n* = 16 paws per group). * *p* < 0.05 vs. Control, # *p* < 0.05 vs. AIA. AIA: antigen induced arthritis; TOFA: tofacitinib; MMP: metalloproteinase

As regards mRNA levels of MMP-1, -3 and -13 at the synovium, an increase in all transcripts was observed in AIA rabbits, which was completely abrogated by the treatment (Fig. 4). No changes were found after the treatment of healthy rabbits with TOFA (data not shown).

### Protein levels of pSTAT1, pSTAT3, SOCS1 and SOCS3 in the synovium of AIA rabbits after TOFA treatment

**Fig. 5:**
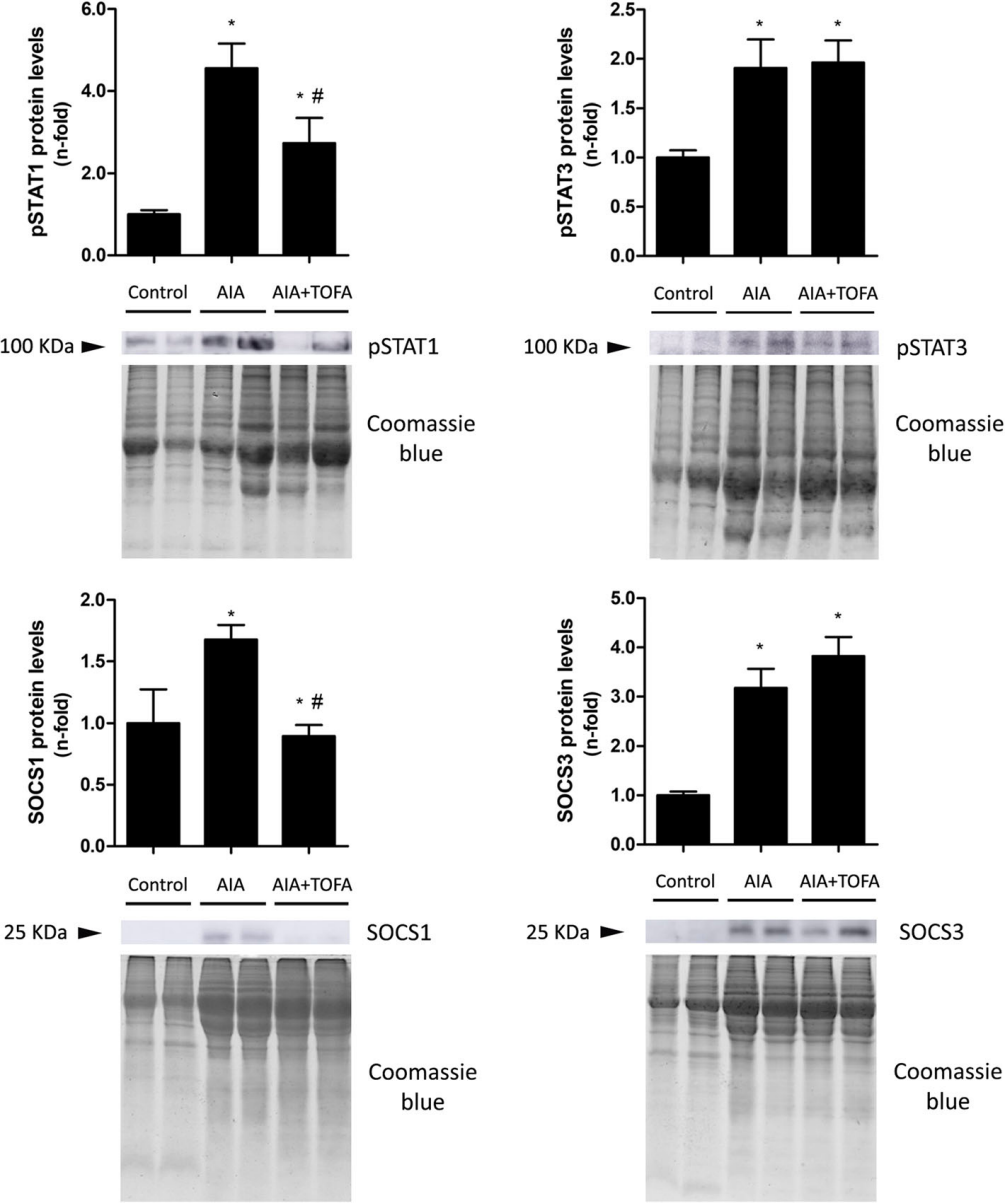
Protein levels of pSTAT1, pSTAT3, SOCS1 and SOCS3 in the synovium of AIA rabbits after TOFA treatment. Western blot analysis of pSTAT1, pSTAT3, SOCS1 and SOCS3 in the rabbit synovium. Data is shown as the mean and SEM (*n* = 16 paws per group). * *p* < 0.05 vs. Control, # *p* < 0.05 vs. AIA. AIA: antigen induced arthritis; TOFA: tofacitinib; STAT: signal transducer and activator of transcription; SOCS: suppressor of cytokine signaling

In order to determine which specific components of the JAK/STAT pathway could account for the beneficial effect observed at the synovium of TOFA-treated AIA animals, both the activation of STAT1 and STAT3, as well as protein levels of their main regulators, SOCS1 and SOCS3, were measured at synovial samples. Rabbits with AIA exhibited a 4.5-fold and a 1.9-fold increase in pSTAT1 and 3, respectively, while levels of SOCS1 and 3 were 1.6-fold and a 3.2-fold higher than levels of controls (*p* = 0.008 and *p* < 0.0001, respectively). With respect to untreated rabbits, TOFA-treated animals showed a fall of 40% in levels of pSTAT1 (*p* = 0.005), and of 46% in levels of SOCS1 (*p* = 0.0001). By contrast, no effect was observed on pSTAT3 or SOCS3 (Fig. 5). TOFA treatment did not have any effect on healthy rabbits (data not shown). Uncropped western blot membranes are provided as Additional file 1.

### Effect of TOFA on NFκB in the synovium of AIA rabbits

**Fig. 6:**
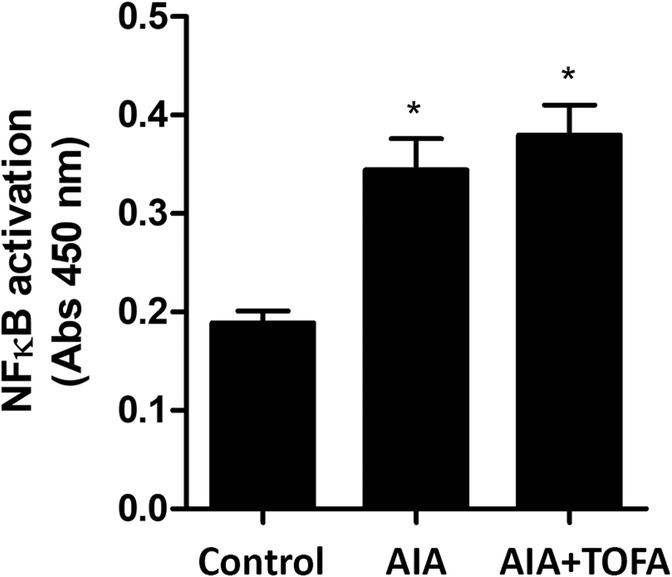
Levels of active NFκB-p65 in the synovium of AIA rabbits after TOFA treatment. Levels of NFκB-p65 able to bind its consensus sequence within the nucleus measured by an enzyme linked immunosorbent assay. Data is shown as the mean and SEM (*n* = 16 paws per group). * *p* < 0.05 vs. Control. AIA: antigen induced arthritis; TOFA: tofacitinib; NF-κB: nuclear factor κB

We next tried to elucidate whether abrogation of JAK/STAT signaling had any early effect on p65-NFκB activation, which may contribute to the prompt benefits of TOFA in the synovium. While a clear activation of the NFκB pathway could be observed in synovial tissues from AIA rabbits at the time of the evaluation, no changes were found in the activity of this pathway as a result of the treatment (Fig. 6). TOFA did not modify NFκB activation in healthy rabbits either (data not shown).

## Discussion

In this experimental approach we have characterized early modifications induced by TOFA at the synovial tissue in a very active RA-like disease. In particular, we could already observe signs of improvement in synovitis histopathology after 2 weeks of treatment. In parallel, there was a downregulation of STAT1-dependent signaling and consequently a fall in its downstream end-products, including SOCS1, MMPs and several pro-inflammatory cytokines. Remarkably, while levels of pSTAT1 were promptly decreased by this JAK inhibitor, those of pSTAT3 remained unchanged.

In agreement with previous observations, our data point to the IFNγ-STAT1 axis as a targetable pathogenic process of rheumatoid synovitis [17, 32]. The activation of this signaling pathway leads to the sustained production of pro-inflammatory cytokines such as TNF and IL6, of which the principal source inside joints are type A and B synoviocytes [14, 33, 34]. Positively, we observed an upregulation of these cytokines along with IFNγ at the synovium of rabbits with AIA, which could be substantially reduced with TOFA. In line with our findings, the drug has been shown to directly suppress the production of IFNγ in human CD4^+^ T cells from patients with RA, an effect which in turn led to a decrease in the synthesis of IL8 by these cells, as well as that of IL6 by synoviocytes [32].

Predictably, these actions occurred in the absence of a noticeable effect on some relevant systemic and local inflammatory processes. In particular, CRP was not lowered with treatment and the macrophage content in the tissues was unchanged, as had already been observed with short TOFA courses in patients with RA [17]. In fact, a clearance of macrophages should not be expected to occur so early in the course of such an active disease, and it would be interesting to seek a shift on the polarization of these cells towards an M2 reparative phenotype, which may be noticeable in the long term. While this possibility was not approached in our study, previous research has suggested that the IFNγ-STAT1 axis is a major driver of M1 polarization [35]. Nonetheless, the exacerbated inflammation at the end of the study, together with the absence of changes in the anti-inflammatory, proregenerative cytokine IL-10 suggests against this possibility in the short term.

At the time studied, active NFκB-p65 was not decreased by the treatment either. Noteworthy, it is known that pSTAT3 can complex with p65, prolonging its transcriptional activity [20, 21], a fact which may also account for the lack of p65 downregulation found in our study. More importantly, our results argue against a crosstalk between STAT1 and NFκB at this early stage of the development of synovitis. Consequently, the observed beneficial effects of TOFA in our experimental model were most probably the sole result of STAT1 signaling blockade, being not related to a general dampening in inflammation. In contrast, studies conducted in psoriatic arthritis (PsA) primary synovial fibroblasts and PsA synovial explants have shown an inhibition of nuclear NFκB translocation at 72 h of incubation with TOFA [18], and similar data were drawn from peripheral blood RA monocytes [19]. Thus, it appears that NFκB inactivation could probably occur later in the course of treatment as a consequence of a global decrease of pro-inflammatory mediators.

Both SOCS1 and 3 were greatly enhanced in the synovium of rabbits with AIA, as had already been found in samples from RA patients [12, 36], possibly reflecting a regulatory response from the injured tissue to counter-balance the effect of MMPs and pro-inflammatory cytokines. In our study, levels of SOCS1 and 3 paralleled those of pSTAT1 and 3, respectively. These findings are consistent with previous observations suggesting that SOCS1 is under STAT1 transcriptional control, whereas SOCS3 mainly depends on STAT3 activation [37–41]. Differential binding preferences of these two factors to pathway components may also contribute to the observed results. While SOCS3 attaches to the cytokine receptor, SOCS1 directly interacts with JAK, as TOFA does, which might lead to a prompt downregulation of SOCS1 in the presence of the JAK inhibitor. To our knowledge, this is the first study assessing the effects of tofacitinib on these regulatory proteins in vivo.

Perhaps the most evident effect of pSTAT1 blockade by TOFA at the rabbit synovial tissue was the potent downregulation of MMP-1, -3 and -13, considering their prominent role in the invasive transformation of the rheumatoid synovium. These matrix-degrading enzymes, which are induced by cytokines such as TNF, IL1β, IL6 and IFNγ [14, 42, 43] are encoded by JAK/STAT responsive genes, as has been reported [44]. In this regard, the potential benefit of JAK inhibitors in preventing the erosive tendency of RA has been little explored. However, it has been found an association between the activation of STAT1 and an up-regulation of MMP-1 and -3 in a subgroup of patients with RA and high grade inflammatory features [45].

## Conclusions

In conclusion, we present here a snap-shot of the effects of TOFA during a flare-up reaction of a RA-like disease. With the aim to focus on mechanisms rather than on efficacy, we were able to dissect the hierarchical network downstream STAT1, as a relevant pathway of inflammation and joint damage independent of NFkB regulation. Overall, our findings suggest that a prompt suppression of STAT1 drives the initial improvement of synovitis in AIA after TOFA treatment and might also account for its efficacy in RA. In addition, our findings point to phospho-STAT1 and MMPs as molecular readouts of an early response to this JAK inhibitor.

## Additional file

Additional file 1: Uncropped western blot membranes. (PDF 130 kb)

## Abbreviations

AIA: Antigen Induced Arthritis
BSA: Bovine Serum Albumin
CRP: C Reactive Protein
DTT: Ditiotreitol
ELISA: Enzyme linked immunosorbent assay
IFN-γ: Interferon Gamma
IgG: Immunoglobulin G
IL: Interleukin
JAK: Janus Kinase Family
MMP: Metalloproteinase
OVA: Ovalbumin
PMSF: Phenylmethanesulfonylfluoride
RA: Rheumatoid Arthritis
RAM11: Rabbit Alveolar Macrophage 11
SOCS: Suppressor of Cytokine Signaling
STAT: Signal Transducer and Activator of Transcription
TNF: Tumor Necrosis Factor
TOFA: Tofacitinib

## References

[1] Iwanawa T, Shikichi M, Kitamura H, Yanase H, Nozawa-Inoue K (2000). Morphology and functional roles of Synoviocytes in the joint. Arch Histol Cytol.

[2] Bartok B, Firestein G (2011). Fibroblast-like synoviocytes: key effector cells in rheumatoid arthritis. Immunol Rev.

[3] Bhattaram P, Chandrasekharan U (2017). The joint synovium: a critical determinant of articular cartilage fate in inflammatory joint diseases. Semin Cell Dev Biol.

[4] Marc Feldmann, Fionula M. Brennan and, Ravinder N. Maini. Role of cytokines in rheumatoid arthritis. Annu Rev Immunol 1996;14:397–440. 10.1146/annurev.immunol.14.1.397 8717520

[5] Andreakos ET, Foxwell BM, Brennan FM, Maini RN, Feldmann M (2002). Cytokines and anti-cytokine biologicals in autoimmunity: present and future. Cytokine Growth Factor Rev.

[6] Brennan FM, McInnes IB (2008). Evidence that cytokines play a role in rheumatoid arthritis. J Clin Invest.

[7] Aittom S, Pesu M (2014). Therapeutic targeting of the JAK / STAT pathway. Basic Clin Pharmacol Toxicol.

[8] Schwartz DM, Bonelli M, Gadina M, Shea JJO (2016). Type I/II cytokines, JAKs, and new strategies for treating autoimmune diseases. Nat Rev Rheumatol.

[9] Mavers M, Rudermand E, Perlman H (2011). Intracellular signal Pathways : potential for therapies. Curr Rheumatol Rep.

[10] Krebs DL, Hilton DJ (2001). SOCS proteins: negative regulators of cytokine signaling. Stem Cells.

[11] Liang Y, Xu W, Peng H, Pan H (2014). SOCS signaling in autoimmune diseases : molecular mechanisms and therapeutic implications. Eur J Immunol.

[12] Isomäki P, Alanärä T, Isohanni P, Lagerstedt a, Korpela M, Moilanen T (2007). The expression of SOCS is altered in rheumatoid arthritis. Rheumatology.

[13] Shouda T, Yoshida T, Hanada T, Wakioka T, Oishi M, Miyoshi K (2001). Induction of the cytokine signal regulator SOCS3/CIS3 as a therapeutic strategy for treating inflammatory arthritis. J Clin Invest.

[14] Malemud CJ (2017). Negative regulators of JAK / STAT signaling in rheumatoid arthritis and osteoarthritis. Int J Mol Sci.

[15] Mahony R, Ahmed S, Diskin C, Stevenson NJ. SOCS3 revisited: a broad regulator of disease, now ready for therapeutic use? Cell Mol Life Sci Springer International Publishing; 2016;73:3323–3336. 10.1007/s00018-016-2234-x PMC11108554 27137184

[16] Ghoreschi K, Jesson M, Li X, Lee J, Ghosh S, Alsup J (2012). Modulation of innate and adaptive immune responses by tofacitinib. J Immunol.

[17] Boyle DL, Soma K, Hodge J, Kavanaugh A, Mandel D, Mease P (2015). The JAK inhibitor tofacitinib suppresses synovial JAK1-STAT signalling in rheumatoid arthritis. Ann Rheum Dis.

[18] Gao W, McGarry T, Orr C, McCormick J, Veale DJ, Fearon U (2016). Tofacitinib regulates synovial inflammation in psoriatic arthritis, inhibiting STAT activation and induction of negative feedback inhibitors. Ann Rheum Dis.

[19] Yarilina A, Xu K, Chan C, Ivashkiv LB (2012). Regulation of inflammatory responses in tumor necrosis factor-activated and rheumatoid arthritis synovial macrophages by JAK inhibitors. Arthritis Rheum.

[20] Grivennikov SI, Karin M (2010). Dangerous liaisons: STAT3 and NF-κB collaboration and crosstalk in cancer. Cytokine Growth Factor Rev.

[21] Lee H, Herrmann A, Deng J-H, Kujawski M, Niu G, Li Z (2009). Persistently activated Stat3 maintains constitutive NF-kappaB activity in tumors. Cancer cell. NIH Public Access.

[22] Schaefer A, Unterberger C, Frankenberger M, Lohrum M, Staples KJ, Werner T (2009). Mechanism of interferon-gamma mediated down-regulation of Interleukin-10 gene expression. Mol Immunol.

[23] Ghoreschi K, Jesson M, Li X, Lee J, Ghosh S, Alsup J (2012). Modulation of innate and adaptive immune responses by Tofacitinib (CP-690,550). J Immunol.

[24] Hodge JA, Kawabata TT, Krishnaswami S, Clark JD, Telliez JB, Dowty ME, et al. The mechanism of action of tofacitinib - an oral Janus kinase inhibitor for the treatment of rheumatoid arthritis. Clin Exp Rheumatol. 2016. 26966791

[25] Pérez-Baos S, Barrasa JI, Gratal P, Larrañaga-Vera A, Prieto-Potin I, Herrero-Beaumont G (2017). Tofacitinib restores the inhibition of reverse cholesterol transport induced by inflammation: understanding the lipid paradox associated with rheumatoid arthritis. Br J Pharmacol.

[26] Buchner E, Bräuer R, Schmidt C, Emmrich F, Kinne RW (1995). Induction of flare-up reactions in rat antigen-induced arthritis. J Autoimmun.

[27] Dowty ME, Jesson MI, Ghosh S, Lee J, Meyer DM, Krishnaswami S (2013). Preclinical to clinical translation of Tofacitinib, a Janus kinase inhibitor, in rheumatoid arthritis. J Pharmacol Exp Ther.

[28] Fujii Y, Sengoku T (2013). Effects of the janus kinase inhibitor CP-690550 (Tofacitinib) in a rat model of oxazolone-induced chronic dermatitis. Pharmacology.

[29] Thacker SG, Abdalrahman Z, Sciumè G, Tsai WL, Anna M (2017). Tofacitinib ameliorates murine lupus and its associated vascular dysfunction. Arthritis Rheumatol.

[30] Krenn V, Morawietz L, Häupl T, Neidel J, Petersen I, König A (2002). Grading of chronic synovitis--a histopathological grading system for molecular and diagnostic pathology. Pathol Res Pract.

[31] Alvarez-Soria MA, Largo R, Santillana J, Sánchez-Pernaute O, Calvo E, Hernández M (2006). Long term NSAID treatment inhibits COX-2 synthesis in the knee synovial membrane of patients with osteoarthritis: differential proinflammatory cytokine profile between celecoxib and aceclofenac. Ann Rheum Dis.

[32] Maeshima K, Yamaoka K, Kubo S, Nakano K, Iwata S, Saito K (2012). The JAK inhibitor tofacitinib regulates synovitis through inhibition of interferon-γ and interleukin-17 production by human CD4+ T cells. Arthritis Rheum.

[33] Marecki S, Riendeau CJ, Liang MD, Fenton MJ (2001). PU.1 and multiple IFN regulatory factor proteins synergize to mediate transcriptional activation of the human IL-1β gene. J Immunol.

[34] Vila-del Sol V, Punzon C, Fresno M (2008). IFN-γ-induced TNF-α expression is regulated by interferon regulatory factors 1 and 8 in mouse macrophages. J Immunol.

[35] Tugal D, Liao X, Jain MK (2013). Transcriptional control of macrophage polarization. Arterioscler Thromb Vasc Biol.

[36] Chan H-C, Ke L-Y, Liu C-C, Chang L-L, Tsai W-C, Liu H-W (2010). Increased expression of suppressor of cytokine signaling 1 mRNA in patients with rheumatoid arthritis. Kaohsiung J Med Sci.

[37] Hong F, Jaruga B, Kim WH, Radaeva S, El-Assal ON, Tian Z (2002). Opposing roles of STAT1 and STAT3 in T cell-mediated hepatitis: regulation by SOCS. J Clin Invest.

[38] De Hooge ASK, Van De Loo FAJ, Koenders MI, Bennink MB, Arntz OJ, Kolbe T (2004). Local activation of STAT-1 and STAT-3 in the inflamed synovium during zymosan-induced arthritis: exacerbation of joint inflammation in STAT-1 gene-knockout mice. Arthritis Rheum.

[39] Snyder M, Huang X-Y, Zhang JJ (2008). Identification of novel direct Stat3 target genes for control of growth and differentiation. J Biol Chem.

[40] Gao B, Wang H, Lafdil F, Feng D (2012). STAT proteins - key regulators of anti-viral responses, inflammation, and tumorigenesis in the liver. J Hepatol.

[41] Satoh JI, Tabunoki H (2013). A comprehensive profile of ChIP-Seq-based STAT1 target genes suggests the complexity of STAT1-mediated gene regulatory mechanisms. Gene Regul Syst Bio.

[42] Vincenti MP, Coon CI, Brinckerhoff CE (1998). Nuclear factor kappaB/p50 activates an element in the distal matrix metalloproteinase 1 promoter in interleukin-1beta-stimulated synovial fibroblasts. Arthritis Rheum.

[43] Bondeson J, Brennan F, Foxwell B, Feldmann M (2000). Effective adenoviral transfer of IkappaBalpha into human fibroblasts and chondrosarcoma cells reveals that the induction of matrix metalloproteinases and proinflammatory cytokines is nuclear factor-kappaB dependent. J Rheumatol.

[44] Araki Y, Wada TT, Aizaki Y, Sato K, Yokota K (2016). Histone methylation and STAT-3 differentially regulate Interleukin-6 – induced matrix metalloproteinase gene activation in rheumatoid arthritis synovial fibroblasts. Arthritis Rheumatol..

[45] Van der Pouw Kraan TCTM, Van Gaalen FA, Kasperkovitz PV, Verbeet NL, Smeets TJM, Kraan MC (2003). Rheumatoid arthritis is a heterogeneous disease: evidence for differences in the activation of the STAT-1 pathway between rheumatoid tissues. Arthritis Rheum.

