# Supplementary figures and images for: Inhibition of pSTAT1 by tofacitinib accounts for the early improvement of experimental chronic synovitis
Source: J Inflamm (Lond). 2019 Jan 29;16:2. doi: 10.1186/s12950-019-0206-2 (PMC6352431; doi:10.1186/s12950-019-0206-2)

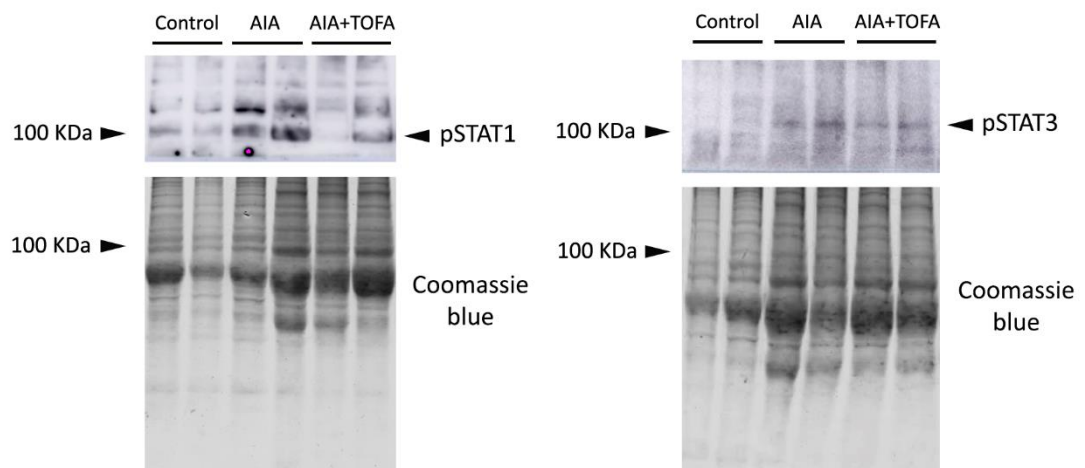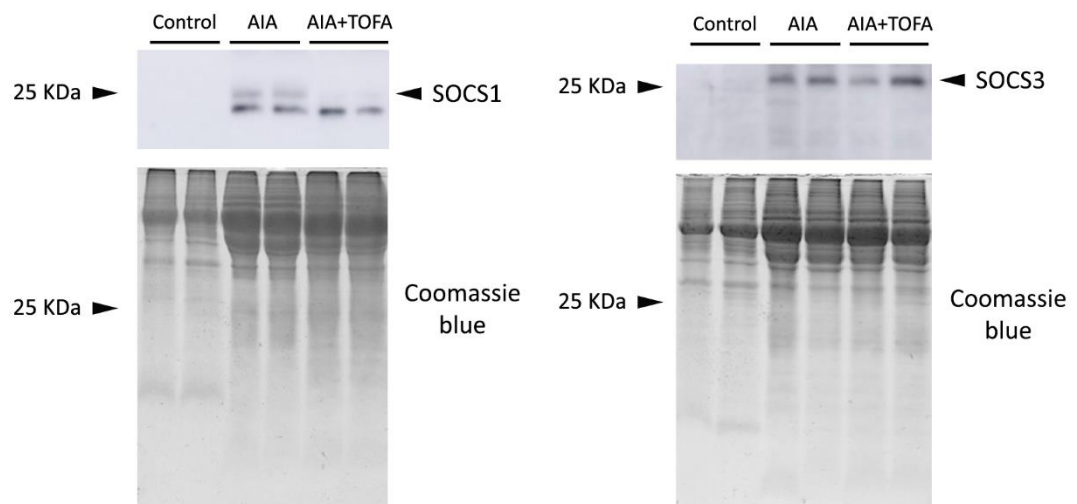

Supplement: Supplementary file 1 — Uncropped western blot membranes. (PDF 130 kb) [file 12950_2019_206_MOESM1_ESM.pdf]
